# On the Trail of Linked Selection

**DOI:** 10.1371/journal.pgen.1006240

**Published:** 2016-08-18

**Authors:** Emily B. Josephs, Stephen I. Wright

**Affiliations:** 1 Department of Evolution and Ecology, University of California, Davis, California, United States of America; 2 Department of Ecology and Evolutionary Biology, University of Toronto, Toronto, Canada; Institute of Science and Technology Austria (IST Austria), AUSTRIA

Distinguishing the relative roles of positive and negative selection along with demographic history in shaping genetic diversity has been a decades-long endeavor. Understanding the forces structuring genetic variation informs us not only about the factors maintaining diversity but also about the fundamental evolutionary parameters that influence natural populations, including the rate and strength of positive selection, the deleterious genetic load experienced by populations, and the factors driving genome evolution. Most attempts at modeling demography, positive selection, or negative selection typically do so by ignoring the contribution of the other forces. Because multiple forces are acting simultaneously, these inferences likely overestimate the role of single evolutionary forces and can lead to biased interpretations. In this issue, Elyashiv et al. [1] make important advances towards addressing this problem by presenting a novel approach to simultaneously estimate the parameters of positive and negative selection based on the spatial patterns of neutral genetic variation and apply this method to *Drosophila*.

Elyashiv et al.’s [1] approach takes advantage of a long-held prediction that patterns of neutral variation can reveal the action of selection at linked sites. Maynard Smith and Haigh first recognized the potential impacts of linked selection in 1974 when they modeled the reduction in neutral genetic diversity that would occur near a site under positive selection, often termed a selective sweep, and argued that this process would reduce diversity genome-wide given sufficiently high rates of positive selection [2]. Support for their theory was first obtained when molecular population genetic data from *Drosophila* showed a correlation between diversity and recombination rate, which was consistent with the idea that selective sweeps are common and have a stronger impact on neutral diversity in regions of low recombination [3,4]. However, Charlesworth and colleagues proposed an alternate explanation: negative selection against the continuous influx of new deleterious mutations (background selection) will also reduce nearby neutral diversity, causing a similar correlation between recombination and genetic diversity [5]. This model has been used to show that many of the major patterns of variation in diversity across the *Drosophila* genome are explainable by this process [6]. Since this alternative mechanism was first proposed, understanding the relative importance of positive and negative selection on linked neutral diversity has been an ongoing challenge in population genetics [7].

Although evidence has continued to mount for the negative relationship between recombination and diversity due to linked selection [8], the expectation that both sweeps and background selection are strongest in regions of low recombination made it difficult to identify what selective forces are responsible for lower diversity in low recombination regions. A number of proposed signals in the data have been thought to be unique predictions that could help distinguish the models, including the allele frequency spectrum in regions of low recombination [3,9] and the exact shape of the relationship between recombination and diversity [10]. However, by broadening background selection predictions to include a class of slightly deleterious mutations, it became clear that many features thought to be unique to positive selection could also be explained by background selection [11]. Until recently, there has been little direct evidence that selective sweeps affect neutral diversity on a genome-wide scale.

**Fig 1:**
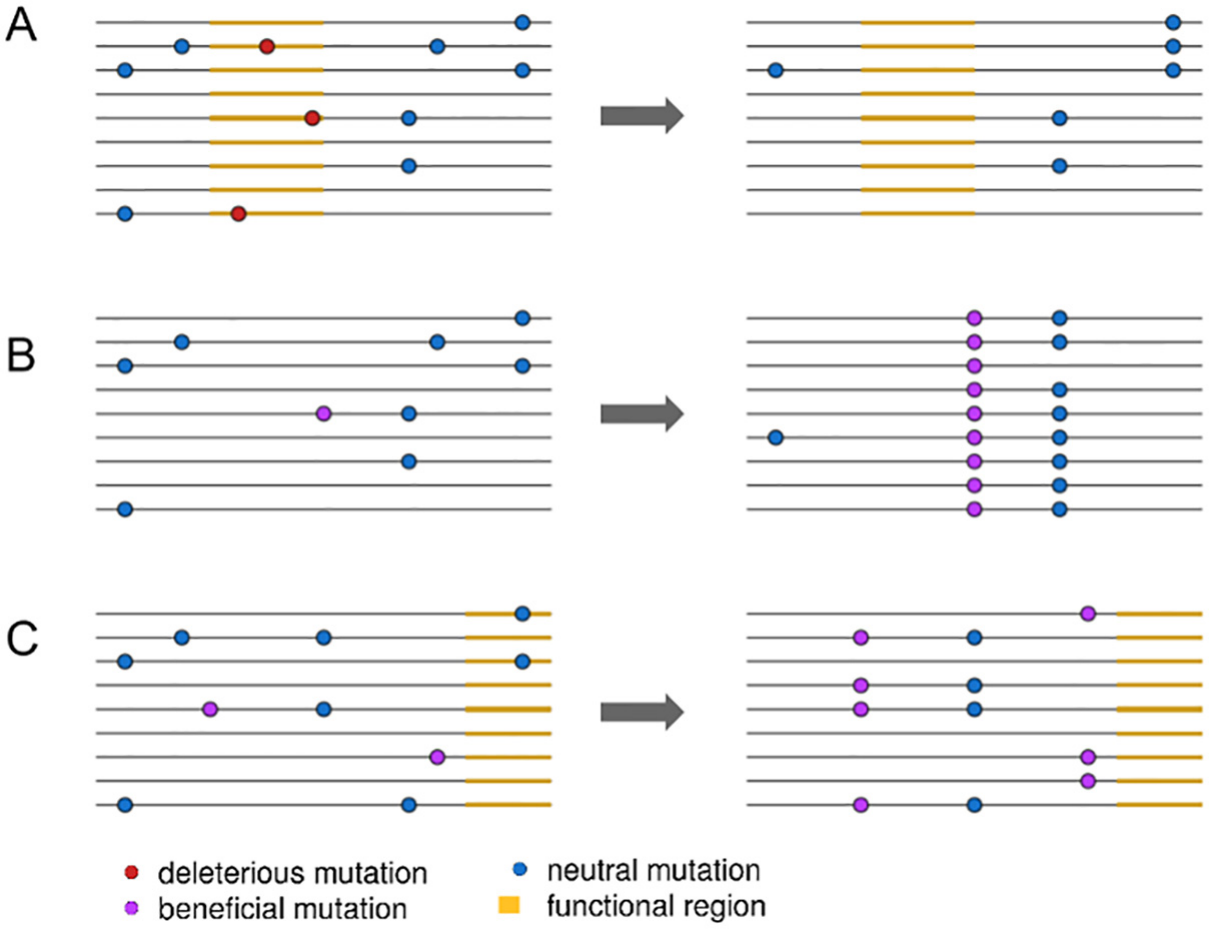
An illustration of three linked selection scenarios. In (A), the removal of deleterious mutations in a functional region, or background selection, reduces nearby linked diversity. In (B), the fixation of a new beneficial mutation by positive selection reduces nearby linked diversity in a process called a selective sweep. In (C), positive selection increases the frequency of two beneficial mutations, reducing diversity but not involving the fixation of any selected mutations.

A key step forward came from the insight that, because selective sweeps result from the fixation of new mutations, fixed substitutions at functional sites provide a unique indicator for the presence of selective sweeps (Fig 1A and 1B) [12,13]. This approach is especially powerful if background selection can be accounted for by comparing to neutral substitutions [14]. For example, neutral diversity is lower around fixed nonsynonymous substitutions than fixed synonymous substitutions in the *Drosophila* genome, providing important evidence that selective sweeps have been common during *Drosophila* adaptation [14]. However, while these patterns were used to infer rates of positive selection, fully quantifying the extent of selective sweeps requires explicitly separating the effects of background selection from sweeps.

Elyashiv et al. [1] used the presence of fixed substitutions to distinguish between sweeps and background selection in a joint modelling framework. Specifically, they model background selection as a function of the density of sites expected to be under purifying selection, such as those in exons and untranslated regions (UTRs). In contrast, they model selective sweeps as a function of the density of fixed substitutions at functional sites. This approach then allows them to estimate deleterious mutation rates directly from data instead of relying on experimentally estimated rates, which often rely on many assumptions and can vary across genotypes [15]. They can then, ultimately, estimate selection coefficients and mutation rates for positively and negatively selected alleles. While Elyashiv et al. [1] are not the first to jointly model the effects of selective sweeps and background selection [16], their approach allows them to generally separate out the effects of sweeps from those of background selection and to do so in a manner that can be applied directly to genomic data.

Using their method, Elyashiv et al. [1] estimate that linked selection reduces diversity by 70%–90% across the genome and that both background selection and selective sweeps contribute in substantial ways to the structuring of genetic diversity. Their joint modeling approach validates previous conclusions that *Drosophila* experiences a high rate of positive selection but also highlights the important role of purifying selection in explaining large-scale patterns of genetic diversity across the genome. These findings cement the view that linked selection is crucial for shaping genomic diversity, so much so that it may be wise to begin including parameters of linked selection—essentially “genomic demography”—in basic population genomic models the same way that standard demographic models are included. Beyond this, approaches based on linked selection may open up new opportunities for investigating the distribution of fitness effects (DFE) of new mutations by allowing us to estimate more precisely the DFE of sites that are so rare they don’t segregate in population genomic samples but do have impacts on linked neutral diversity.

Although Elyashiv et al. [1] have made significant progress in our understanding of linked selection, there are still some avenues for improvement. Their method is dependent on knowledge of functional sites that accurately predict where purifying and positive selection act in the genome, and, as they note, selection outside of these regions will affect their predictions. Genes and genomic regions also differ in their fraction of selectively constrained sites, and so explicitly incorporating information about cross-species divergence into models of purifying selection [17] may also help refine the model parameters.

More crucially, while conditioning their inference of selective sweeps compared to background selection on the presence of fixed substitutions, other forms of positive selection that do not involve fixations, such as polygenic adaptation, are likely parameterized as background selection (Fig 1C). If polygenic adaptation is common [18], this should incorrectly increase the strength of background selection inferred in the model. Recent work in humans implies that large fractions of the genome may in fact be subject to directional selection on quantitative traits [19], further indicating that polygenic selection can have genome-wide effects. Indeed, Elyashiv et al. [1] estimate a much higher deleterious mutation rate than appears plausible given estimates from the literature, suggesting an important role for polygenic adaptation in driving down diversity near functional sites. Developing techniques to properly account for polygenic adaptation will not only improve accuracy for inferring selection parameters and the effects of linked selection but also provide further insights into the extent to which positive selection influences the structuring of genetic diversity genome-wide.

Beyond extending linked selection approaches to better deal with poor annotations and polygenic adaptation, estimating the effects of linked selection in additional species beyond *Drosophila* holds great promise. While patterns of linked selection generally differ between *Drosophila* and other species, the causes of these differences remain uncertain [8]. The test developed by Sattath and colleagues [14] to detect selective sweeps by comparing diversity around functional substitutions to diversity around nonfunctional substitutions has since been applied to additional species, including humans, mice, maize, and *Capsella grandiflora* [20–23], with mixed evidence for recurrent sweeps. However, this method’s use of synonymous substitutions as a neutral control may not be effective in larger genomes, in which nonsynonymous substitutions may be more common in regions of low constraint, reduced background selection, and higher neutral diversity, masking the signals of selective sweeps [24]. Because Elyashiv et al.’s [1] method can use the distribution of selected sites present in the actual genome under investigation, it may be more robust to variation in constraint across the genome and be useful for investigating linked selection in large genomes.

Patterns of linked selection are also likely to differ among species of varying population sizes. There is already evidence that the effects of linked selection on neutral diversity increase with species census size [25], and Elyashiv et al.’s [1] joint modelling approach may help tease apart why these effects vary with effective population size (Ne). For example, joint modelling approaches could help improve the parameterization of positive selection in species with smaller Ne, in which signals may be weaker due to lower rates of positive selection. However, we may also expect a larger proportion of adaptation in species with high Ne to involve multiple alleles, altering our expectations of how linked selection will shape nearby neutral diversity [26,27]. This further highlights the need to develop approaches that independently parameterize polygenic adaptation, although this remains a difficult challenge.

Furthermore, the relative importance of linked selection on neutral diversity may vary with mating system [5,28]. Reduced effective recombination rates in selfing species are expected to play an important role in limiting both the removal of deleterious alleles by purifying selection [29,30] and the fixation of adaptive alleles due to positive selection [31,32], but this is difficult to accurately quantify. Intriguingly, there is evidence that there is a much larger drop in genome-wide diversity in selfers than in outcrossers due to the strong effects of linked selection [33]. However, the effects of linked selection may be especially difficult to investigate in selfers because low recombination will mean that subsequent events of linked selection mask those that occurred previously. Joint modelling approaches may help investigate these patterns and possibly help quantify the extent to which positive and purifying selection are reduced in selfing lineages.

Overall, the comprehensive model of linked selection developed by Elyashiv et al. [1] both reinforces the growing consensus that linked selection is an important contributor to genetic variation and provides a useful framework for investigating not only the specific impacts of linked selection but also the general selective parameters that shape genomic variation. As we have outlined above, their work opens up a number of further opportunities to investigate the action of selection across a genome and provides an important step forward in the ongoing challenge of quantifying the relative importance of different evolutionary forces.
